# Clinical application of anterior ring internal fixator system combined with sacroiliac screw fixation in Tile C pelvic fracture treatment

**DOI:** 10.1186/s13018-021-02863-y

**Published:** 2021-12-14

**Authors:** Lin Liu, Shicai Fan, Donggui Zeng, Yuhui Chen, Hui Song, Letian Zeng, Dadi Jin

**Affiliations:** 1 Orthopedic Trauma, University of Chinese Academy of Sciences Shenzhen Hospital, Shenzhen, Guangdong People’s Republic of China; 2 The Third Affiliated Hospital, Southern Medical University, Guangzhou, Guangdong People’s Republic of China

**Keywords:** Anterior ring internal fixator system, Iliosacral screw, Pelvic fracture, Internal fixation, Minimal invasiveness

## Abstract

**Background:**

How to perform minimally invasive surgery for Tile C pelvic fracture is a major problem in clinical practice. We performed minimally invasive surgery for Tile C pelvic fracture using anterior ring internal fixator systems combined with sacroiliac screw fixation.

**Objective:**

To investigate the advantages and efficacy of anterior ring internal fixator systems combined with sacroiliac screw fixation in the treatment of Tile C pelvic fracture.

**Methods:**

From May 2017 to May 2020, 27 patients with Tile C pelvic fracture who underwent anterior ring internal fixator system combined with sacroiliac screw fixation (group A) and 21 patients with Tile C pelvic fracture who underwent plate-screw system combined with sacroiliac screw fixation (group B) were retrospectively analyzed.

**Results:**

All 48 patients were followed up for more than 12 months, all fractures healed within 3–6 months. The operative time, intraoperative bleeding volume, blood transfusion volume, incision length, hospital stay, complication rate and Majeed score were 63.5 ± 10.7 min, 48.3 ± 27.9 ml, 0 ml, 4.5 ± 0.8 cm, 10.2 ± 2.7 d, 3.7% and 89.7 ± 4.6 points, respectively, in group A and 114.8 ± 19.1 min, 375 ± 315.8 ml, 266.7 ± 326.6 ml, 9.2 ± 3.9 cm, 20.9 ± 5.7 d, 23.8% and 88.7 ± 4.9 points, respectively, in group B. Combined excellent and good rates of the Matta evaluation and Majeed score were 100% in both groups. There were no significant differences in the Matta evaluation or Majeed score between the two groups (both *P* > 0.05), whereas the operative time, intraoperative bleeding volume, blood transfusion volume, incision length and hospital stay were significantly less in group A (all *P* < 0.05).

**Conclusion:**

An anterior ring internal fixator system combined with sacroiliac screw fixation can effectively treat Tile C pelvic fracture, and has advantages, including minimal invasiveness, simple operation, short operative time, safe and reliable features, fewer complications, short hospital stay and a good curative effect.

## Introduction

Tile C pelvic fracture is an extremely severe pelvic injury, which is difficult to treat in the early stage. The traditional treatment methods for Tile C pelvic fracture include external fixation—with a fixed frame or open reduction and plate-screw fixation, which have disadvantages such as poor stability, inconvenient daily care, difficult exposure, large trauma and many complications, respectively. With the development of minimally invasive technology, combined closed reduction and percutaneous sacroiliac screw fixation has become a common-such surgery for posterior pelvic ring injury. The subcutaneous internal fixator [1, 2] or INFIX technology [3–5] has also been gradually increasingly used in clinical practice as a minimally invasive surgery for anterior pelvic ring injury and has achieved good results. However, minimally invasive surgical treatment for Tile C pelvic fracture is rarely used at present. It remains one of the biggest challenges for orthopedic trauma surgeons to perform minimally invasive surgery for Tile C pelvic fractures in the early stage. To explore appropriate minimally invasive surgical treatment for Tile C pelvic fractures, Since May 2017, we have used an anterior ring internal fixator system combined with sacroiliac screw fixation for the minimally invasive treatment of Tile C pelvic fracture, have achieved satisfactory results.

## Materials and methods

### Clinical data

#### Inclusion and exclusion criteria

Inclusion criteria: ① Tile C pelvic fracture, ② Time to surgery after injury < 45 d, ③ age > 18 years and < 70 years. Exclusion criteria: ① patients with severe cardiopulmonary, hepatic and renal incompetence or coagulation dysfunction, ② open pelvic fractures requiring emergency management, ③ systemic or local infection, ④ combined with severe acetabular fracture, ⑤ combined with major vascular injuries.

#### General information

From May 2017 to May 2020, 27 patients with Tile C pelvic fracture (group A) were treated with an anterior ring internal fixator system combined with sacroiliac screw fixation, including 19 males and 8 females, with a mean age of 46.6 years (32–58 years). A further 21 patients with Tile C pelvic fracture (group B) were treated with a plate-screw system combined with sacroiliac screw fixation, including 15 males and 6 females, with a mean age of 47.1 years (22–68 years). There was no significant difference in gender, age, cause of injury, fracture type or combined injury between the two groups (all *P* > 0.05) (Tables 1, 2).

**Table 1:** General patient information

Group	Case	Age (years)	Sex	Cause	Tile classification
		$\overline{X}$ ± *S*	M:F	Accident:Falling:Bruised	C1:C2:C3
A	27	46.6 ± 7.6	19:8	12:11:4	10:11:6
B	21	47.1 ± 15.6	15:6	9:8:4	7:9:5
*P* value		0.884	0.938	0.133	0.814

**Table 2:** Accompanying injuries of the patients

Group	Case	1	2	3	4	5	6	7	8
A	27	7	17	13	8	8	3	2	2
B	21	7	15	11	9	4	4	3	2
*P* value		0.585	0.547	0.777	0.352	0.450	0.412	0.450	0.798

(1. Lumbosacral plexus injury; 2. four-limb fracture; 3. lumbar fracture, 4. rib fracture, 5. brain injury, 6. neurovascular injury in lower limbs; 7. bladder rupture, 8. colon rupture.)

### Surgical method

All patients underwent general anesthesia with endotracheal intubation and lay in a supine position on a special X-ray-transparent operating table.

#### Surgical method in group A

The patients underwent closed reduction of the fracture. For the patients in whom it was difficult to perform closed reduction of fracture, a Starr frame was used to assist in fracture reduction. For the patients in whom it was impossible to perform closed reduction of the fracture, a lateral rectus abdominis approach was used for the exploration and release of the lumbosacral plexus nerves, reduction of a sacral fracture or reduction of a sacroiliac joint dislocation. All patients were firstly treated with percutaneous sacroiliac screw fixation, and then with internal fixator system fixation of the anterior ring fracture.

##### Sacroiliac screw placement

**Fig. 1:**
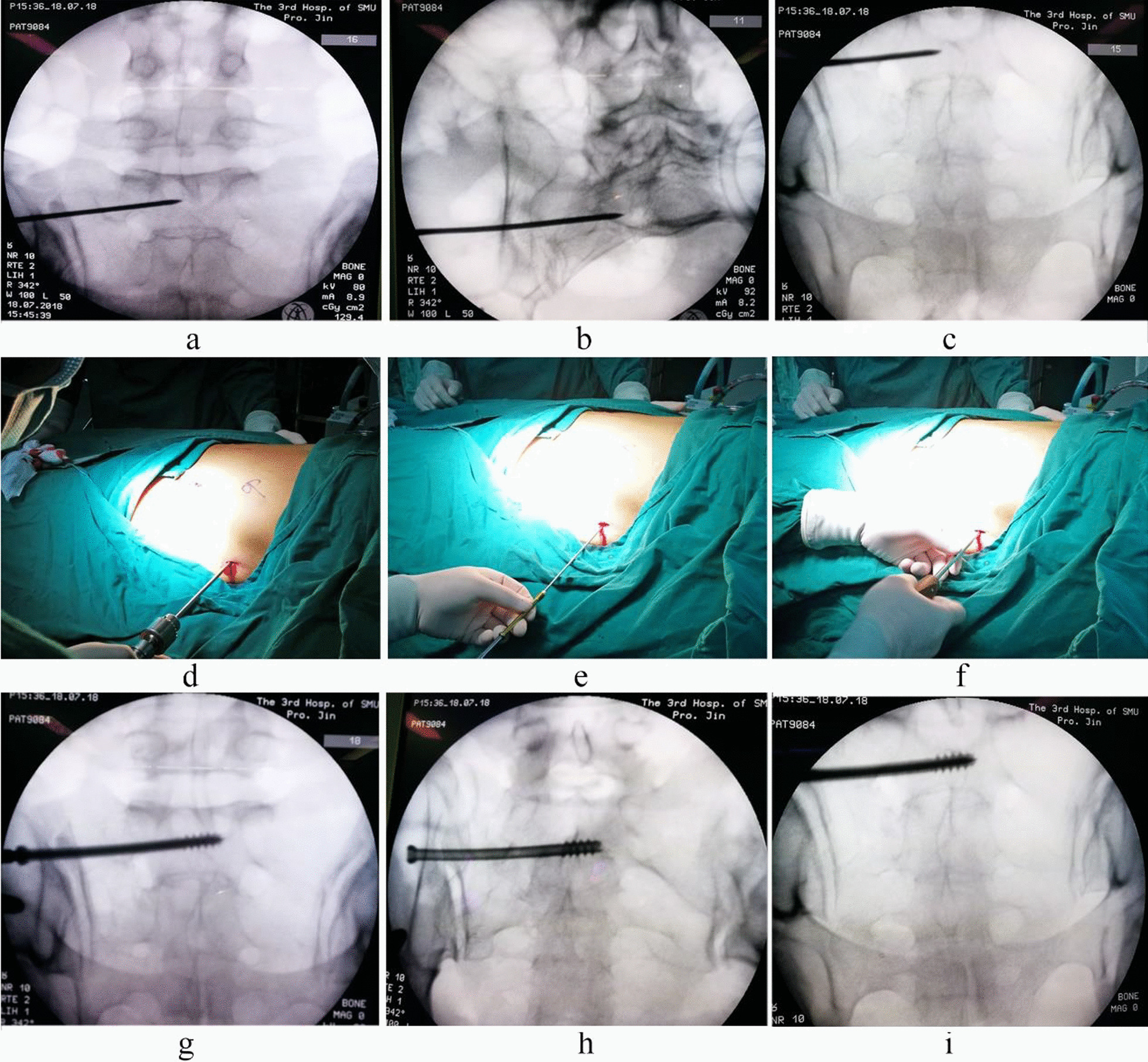
Sacroiliac screw placement. (A 36-year-old man with anterior and posterior pelvic ring injuries caused by a fall. **a**–**c** Fluoroscopic images of guide needle placement, **a** anteroposterior view, **b** inlet view, **c** outlet view; **d**–**f** sacroiliac screw placement: **d** drilling, **e** screw placement, **f** completed screw placement; **g**–**i** fluoroscopic images of the placed sacroiliac screw, **g** anteroposterior view, **h** inlet view, **i** outlet view.)

A C-arm X-ray machine was used for fluoroscopic pelvis positioning (pelvic anteroposterior, inlet and outlet views). Once it was observed that the fracture reduction was satisfactory, a guide needle with a 2.5-mm-diameter was slowly struck with a hammer on the iliac bone at the intersection of the middle one-third and posterior one-third of a line connecting the anterior and posterior superior iliac spines, the tail of the guide needle was tilted upward approximately 10° and backward approximately 20°, and the guide needle was slowly passed through the iliac bone and sacroiliac joint to reach the S1 vertebral body. Pelvic fluoroscopy (anteroposterior, inlet and outlet views) was performed to observe the position of the guide needle, determine the correct position of the guide needle and determine the length of the required screw (Fig. 1a–c). Subsequently, a hollow sacroiliac screw of 70–100-mm-length and 7.3-mm-diameter (Shandong Weigao Company, Yantai, China) was screwed in Fig. 1d–f. When the patient had bilateral sacroiliac joint injuries, a sacroiliac screw was inserted on the other side by the same method, and fluoroscopy was performed to determine the length and position of the screw and fracture reduction (Fig. 1g–i).

##### Placement of the anterior ring internal fixator system

**Fig. 2:**
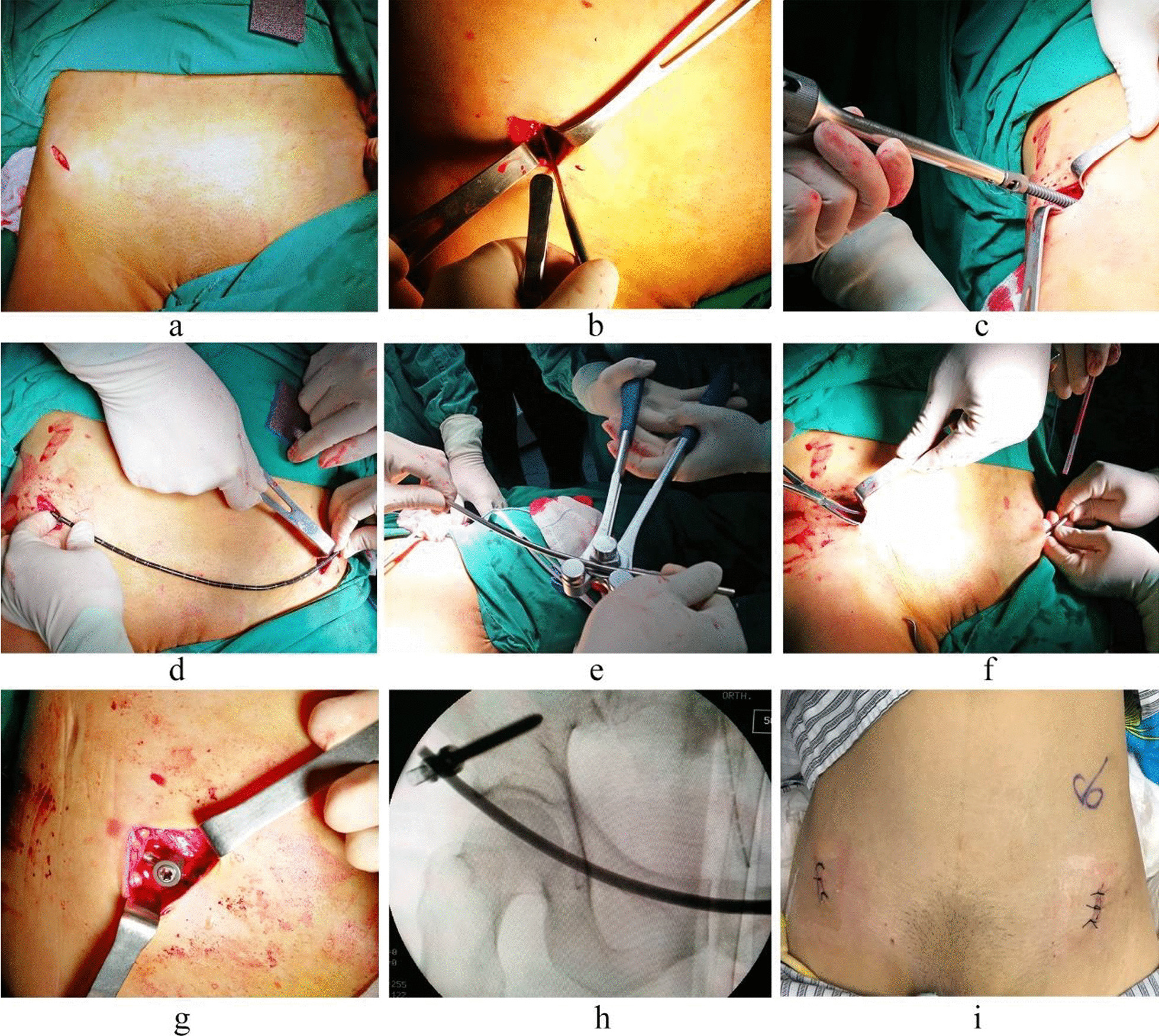
Surgical operation for anterior ring internal fixator system fixation. (A 36-year-old man with anterior and posterior pelvic ring injuries caused by a fall. **a** Incision, **b** cortical bone opening, **c** screw placement, **d** connecting rod die, **e** pre-bending of a connecting rod, **f** placement of the connecting rod through the subcutaneous channel, **g** connecting the rod fixation, **h** fluoroscopic image of the connecting rod, **i** postoperative incision.)

Taking the anterior inferior iliac spine as the center, a short oblique incision (2–3 cm) was made along the direction of the inguinal ligament (Fig. 2a). The intermuscular space between the sartorius muscle and the tensor fasciae latae was bluntly separated to the anterior inferior iliac spine, paying attention to protect the lateral femoral cutaneous nerve. A 2.5-mm-diameter guide needle was inserted from approximately 5 mm above the superior edge of the anterior inferior iliac spine in the direction of posterior superior iliac spine. The guide needle angle with respect to the coronal, sagittal and horizontal planes was approximately 56°, 26° and 65°, respectively, in men, and 51°, 25° and 59°, respectively, in women. According to the position and open end direction of the guide needle (Fig. 2b), a screw passage was established between the internal and external iliac plates, and its depth was measured. A screw with 7.5-mm-diameter and a suitable length (60–100 mm, Xiamen Dabo Medical Instrument Co., Ltd., China) was inserted for fixation of the universal titanium alloy bracket (Fig. 2c). The tail of the screw protruding out of the bone was 10–15 mm in length, and the screw was inserted in the contralateral side using the same method. When it was required to place a fixation screw in the pubic region, an approximately 2–3 cm transverse incision was made in the pubic symphysis region, the pubic symphysis region was exposed, and a universal fixation screw of 4.5 mm diameter and 35–45 mm length was inserted vertically (from up to down) into the pubis surface, being located approximately 1-cm-lateral to the middle of pubic symphysis. The connecting rod die was pre-bent forward and downward, and its length was according to the abdominal shape (Fig. 2d). A 6-mm-diameter titanium rod was pre-bent and cut off according to the shape of the connecting rod die (Fig. 2e), and a subcutaneous channel on the deep fascia was established [3, 5]. The titanium rod was passed through the channel and placed on the fixation screws to be locked and fixed (Fig. 2f–h). The distance between the titanium rod and the bone surface was 20–25 mm, and the length between the two ends of the titanium rod and the fixation screw was approximately 5 mm. Finally, the incision was sutured (Fig. 2i).

##### Typical cases

**Fig. 3:**
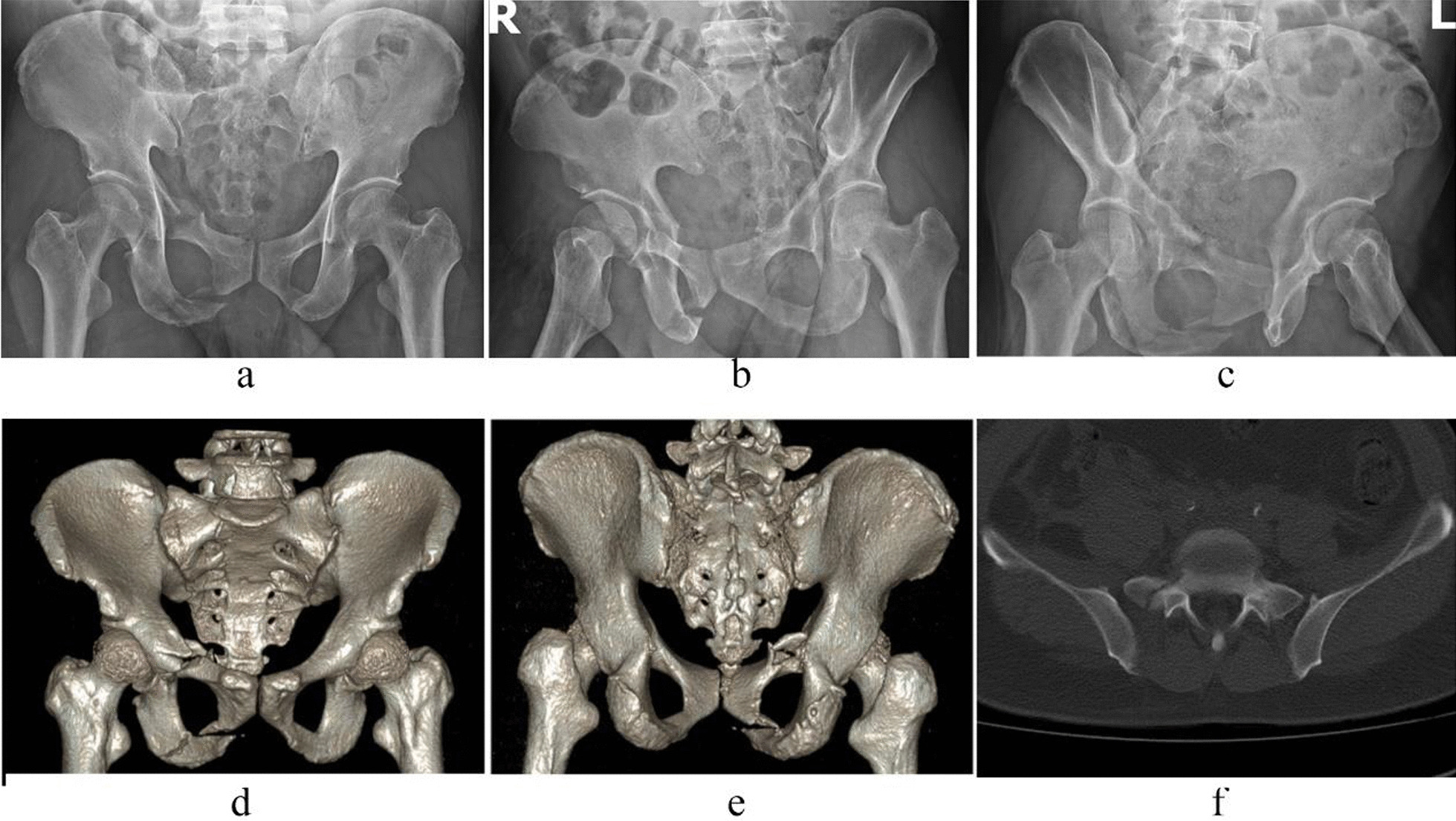
Preoperative X-ray and CT images of case 1. (**a** Pelvic anteroposterior X-ray image, **b** iliac oblique position X-ray image, **c** obturator oblique position X-ray image, **d**, **e** preoperative CT 3D reconstruction image, **f** CT horizontal slice image.)

**Fig. 4:**
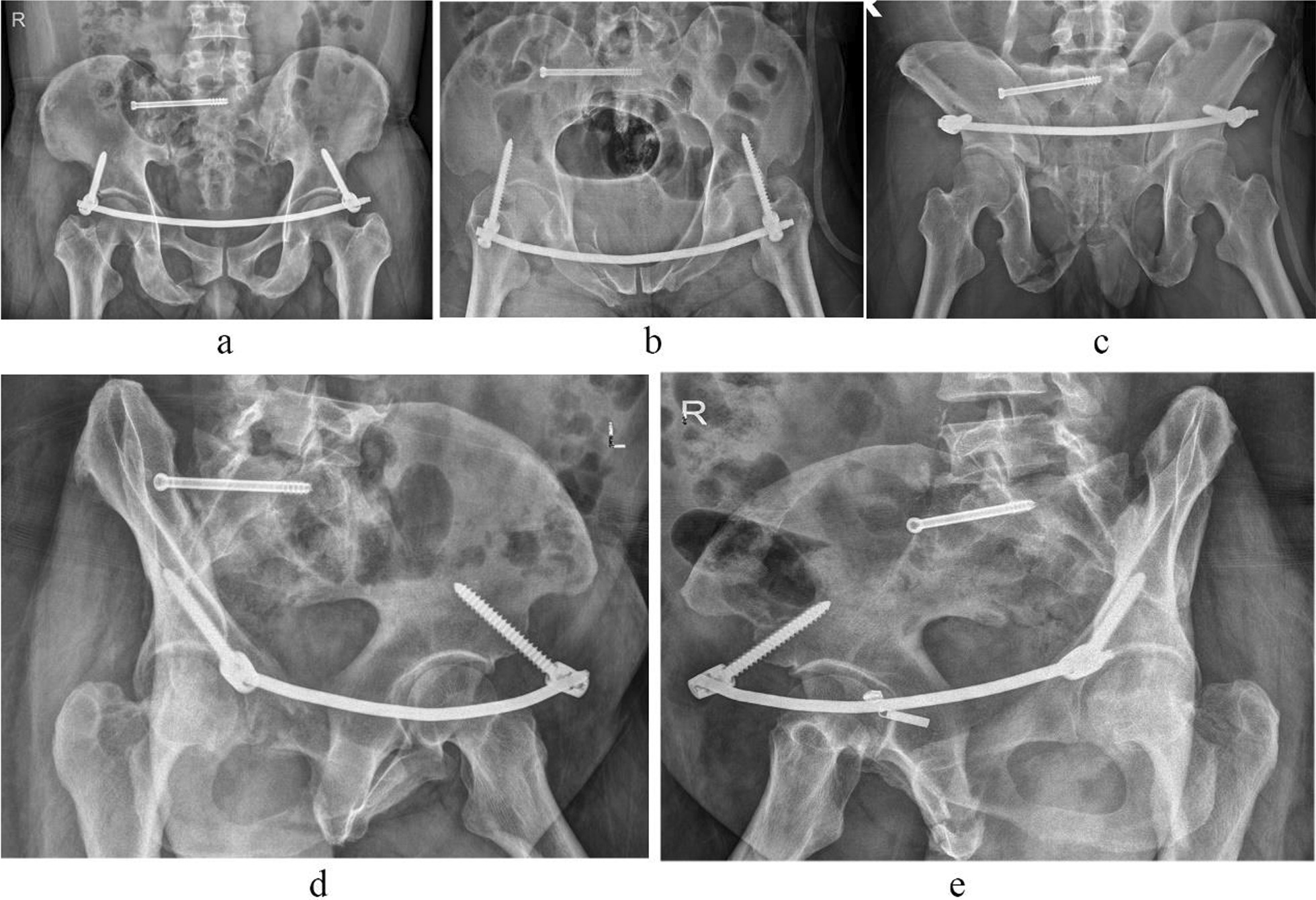
Postoperative images of case 1. (**a** Pelvic anteroposterior X-ray image, **b** pelvic inlet X-ray image, **c** pelvic outlet X-ray image, **d** obturator oblique position X-ray image, **e** iliac oblique position X-ray image.)

Case 1, Yuan XX, a 52-year-old male patient, was admitted to hospital due to a falling injury 3 days earlier. Diagnosis was a pelvic fracture (Tile C1) combined with multiple fractures in the right distal radius and carpus, and a transverse process fracture at the level of L5. Preoperative images are shown in Fig. 3. On the fifth day after injury, the patient underwent closed reduction of the pelvic fracture, including percutaneous cannulated screw internal fixation for the posterior pelvic ring fracture and two-screw internal fixator system fixation for the anterior pelvic ring fracture. The operation duration was 50 min, with a 20 ml bleeding volume. The patient was discharged after 9 days of hospitalization. Postoperative images are shown in Fig. 4.

**Fig. 5:**
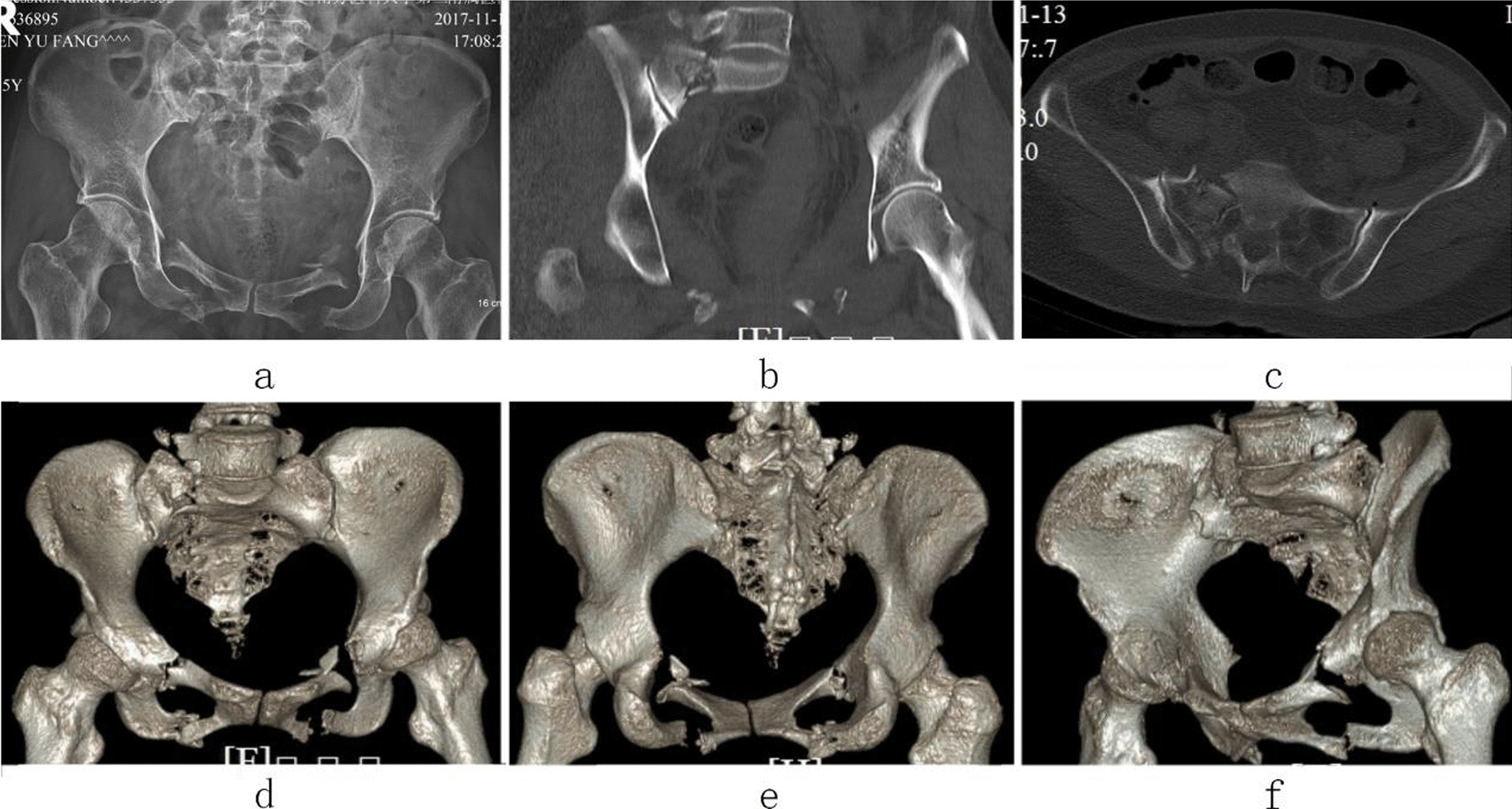
Preoperative images of case 2. (**a** Pelvic anteroposterior X-ray image, **b** CT sagittal image, **c** CT horizontal slice image, **d**–**f** CT 3D reconstruction images.)

**Fig. 6:**
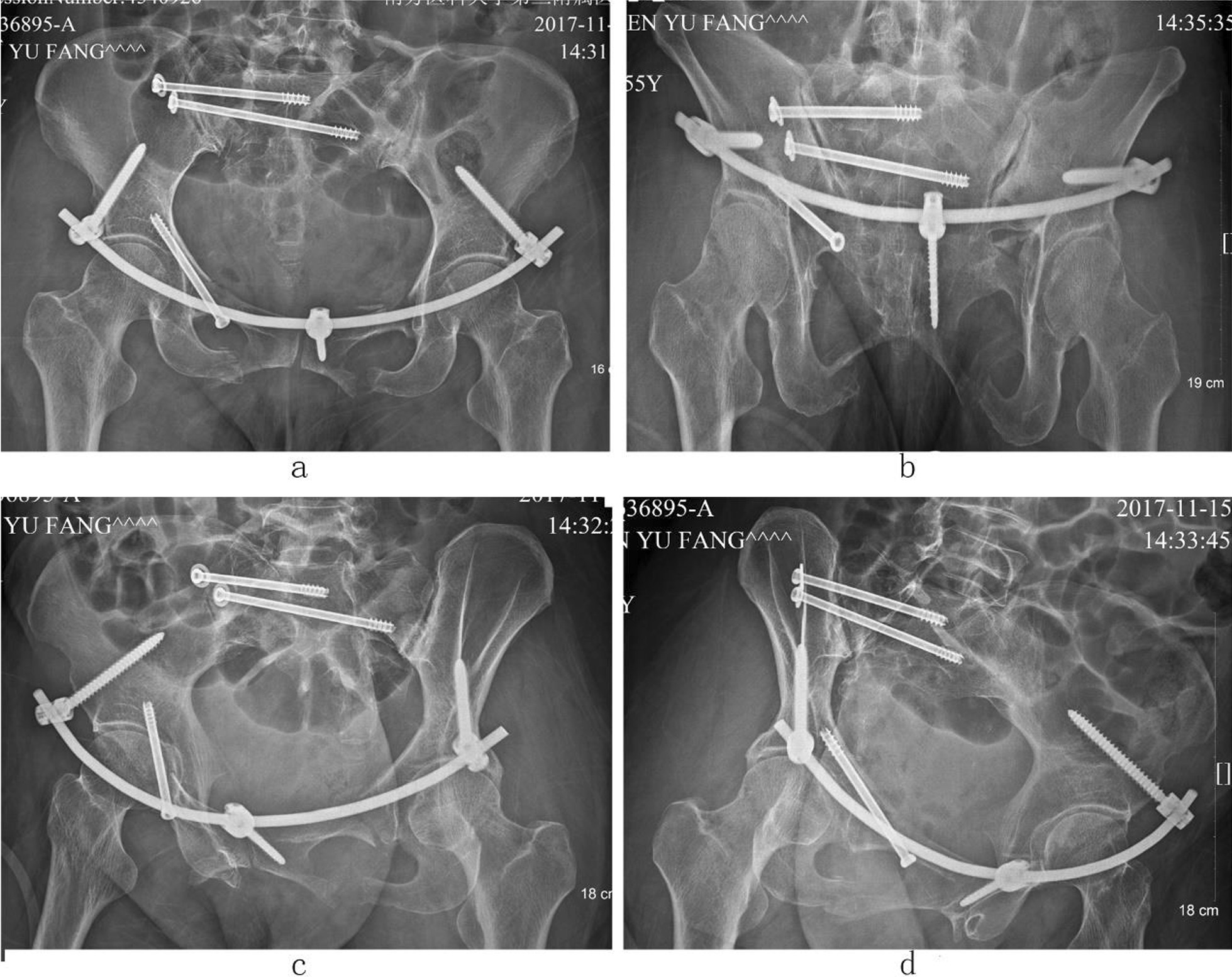
Postoperative X-ray images of case 2. (**a** Pelvic anteroposterior X-ray image, **b** pelvic outlet X-ray image, **c** iliac oblique position X-ray image, **d** obturator oblique position X-ray image.)

**Fig. 7:**
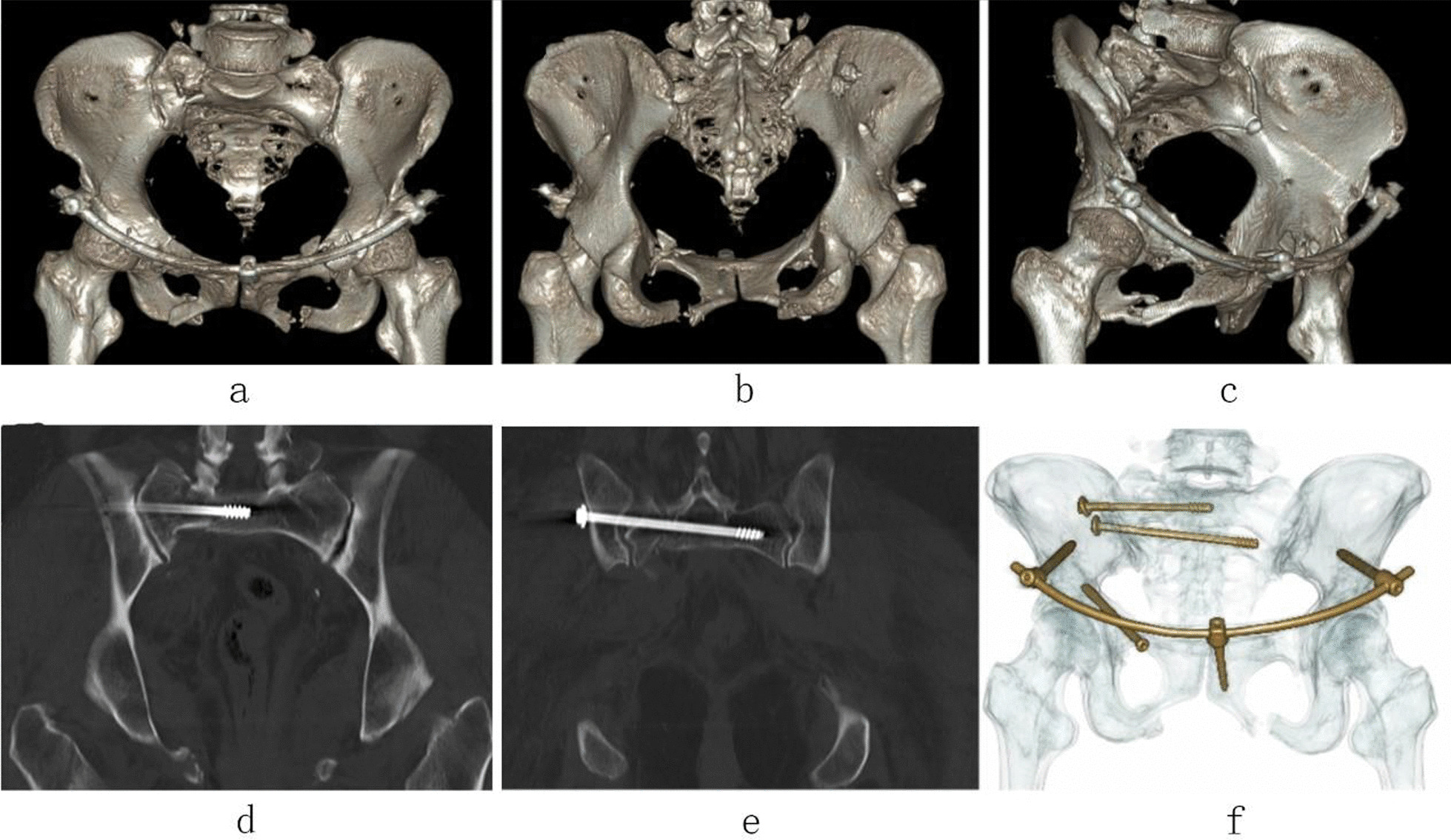
Postoperative CT images of case 2. (**a**–**c**, **f** CT 3D reconstruction images, **d**, **e** CT sagittal image.)

Case 2, Ren XX, a 55-year-old female patient, was admitted to hospital because of a car crash injury 3 days earlier. Diagnosis was a pelvic fracture (Tile C2) combined with cerebral concussion and multiple soft tissue contusions throughout the body. Preoperative images are presented in Fig. 5. On the sixth day after injury, the patient underwent closed reduction of the pelvic fracture, percutaneous cannulated screw internal fixation with percutaneous cannulated screws for the posterior pelvic ring fracture and three-screw internal fixator system fixation for the anterior pelvic ring fracture. The operation duration was 60 min, with a 20 ml bleeding volume. Postoperative images are shown in Figs. 6 and 7. The patient was discharged after 12 days of hospitalization and followed up for 20 months. The Majeed functional score at the final follow-up was 92 points.

**Fig. 8:**
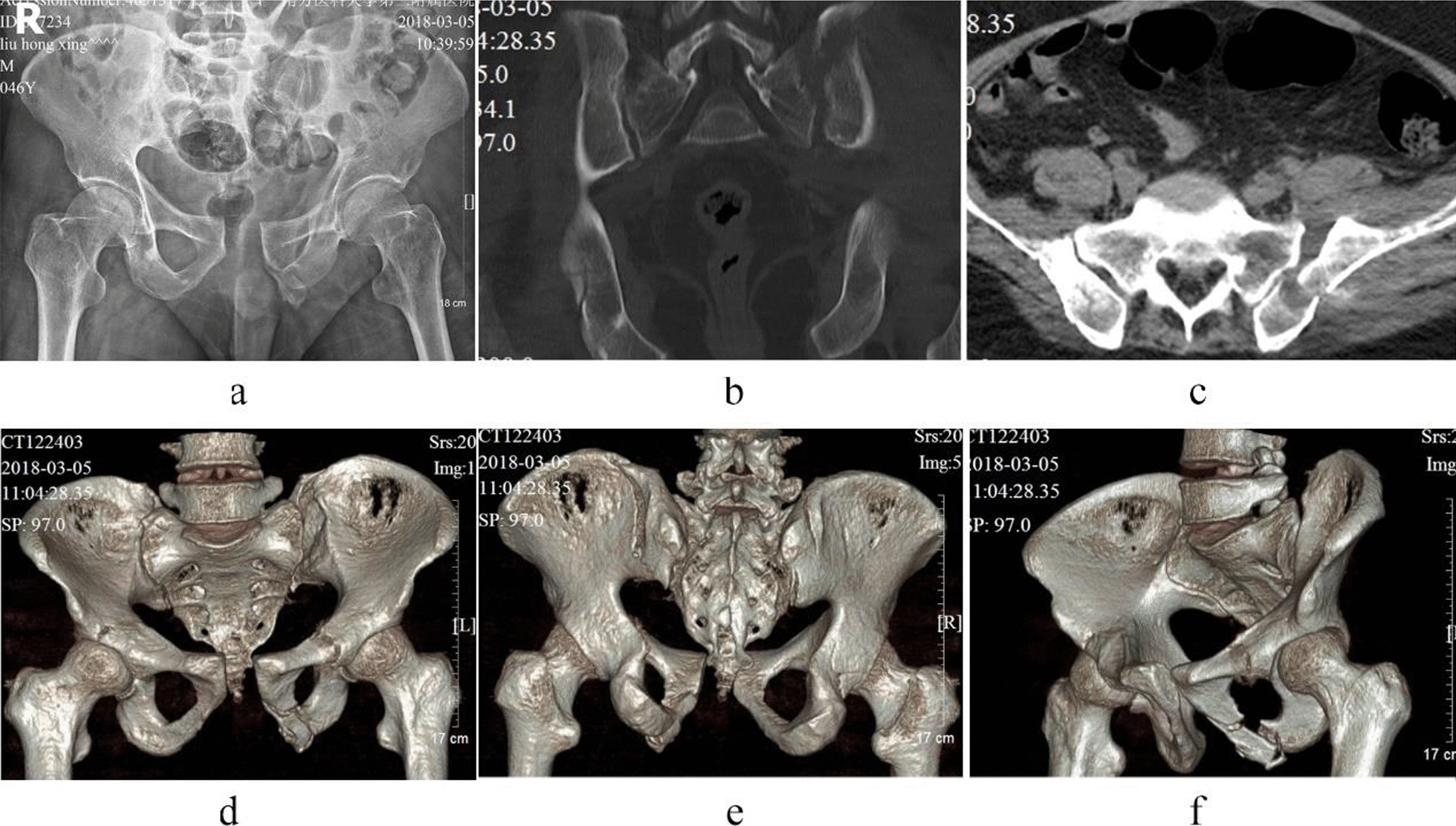
Preoperative images of case 3. (**a** Pelvic anteroposterior X-ray image, **b** CT sagittal image, **c** CT horizontal slice image, **d**–**f** CT 3D reconstruction images.)

**Fig. 9:**
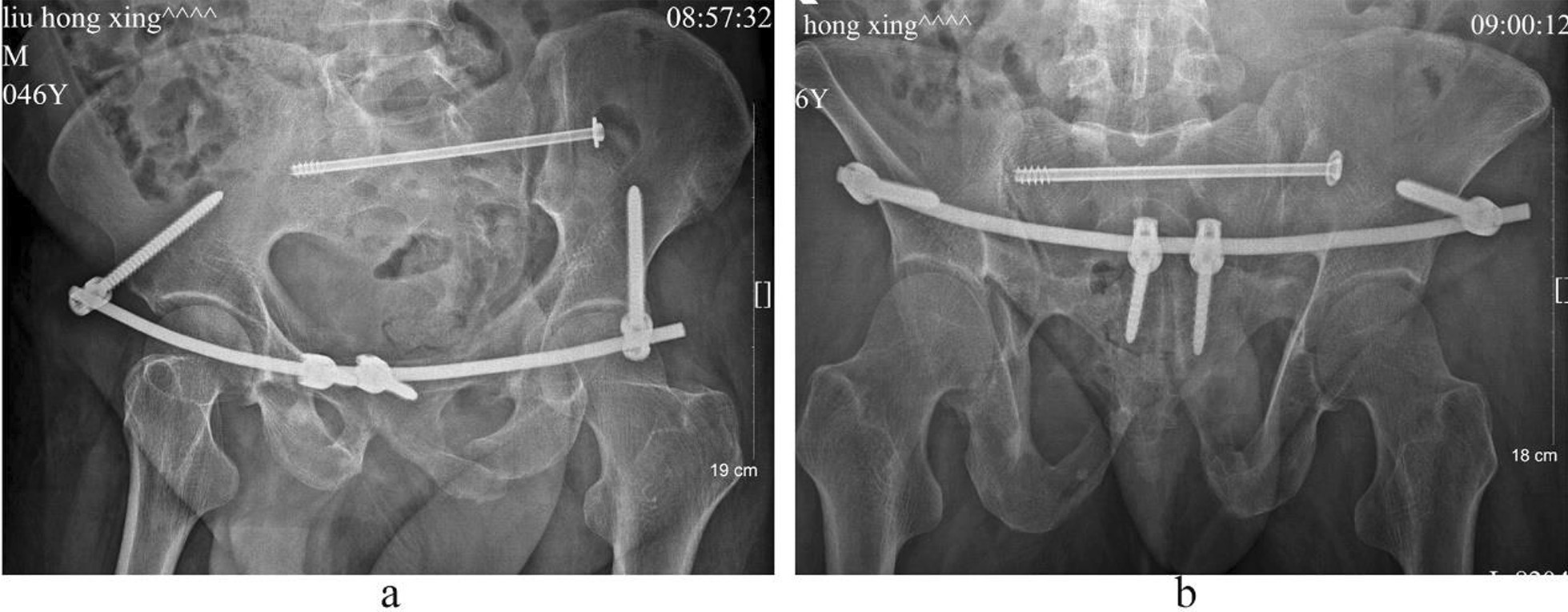
Postoperative X-ray images of case 3. (**a** Pelvic anteroposterior X-ray image, **b** pelvic outlet X-ray image.)

**Fig. 10:**
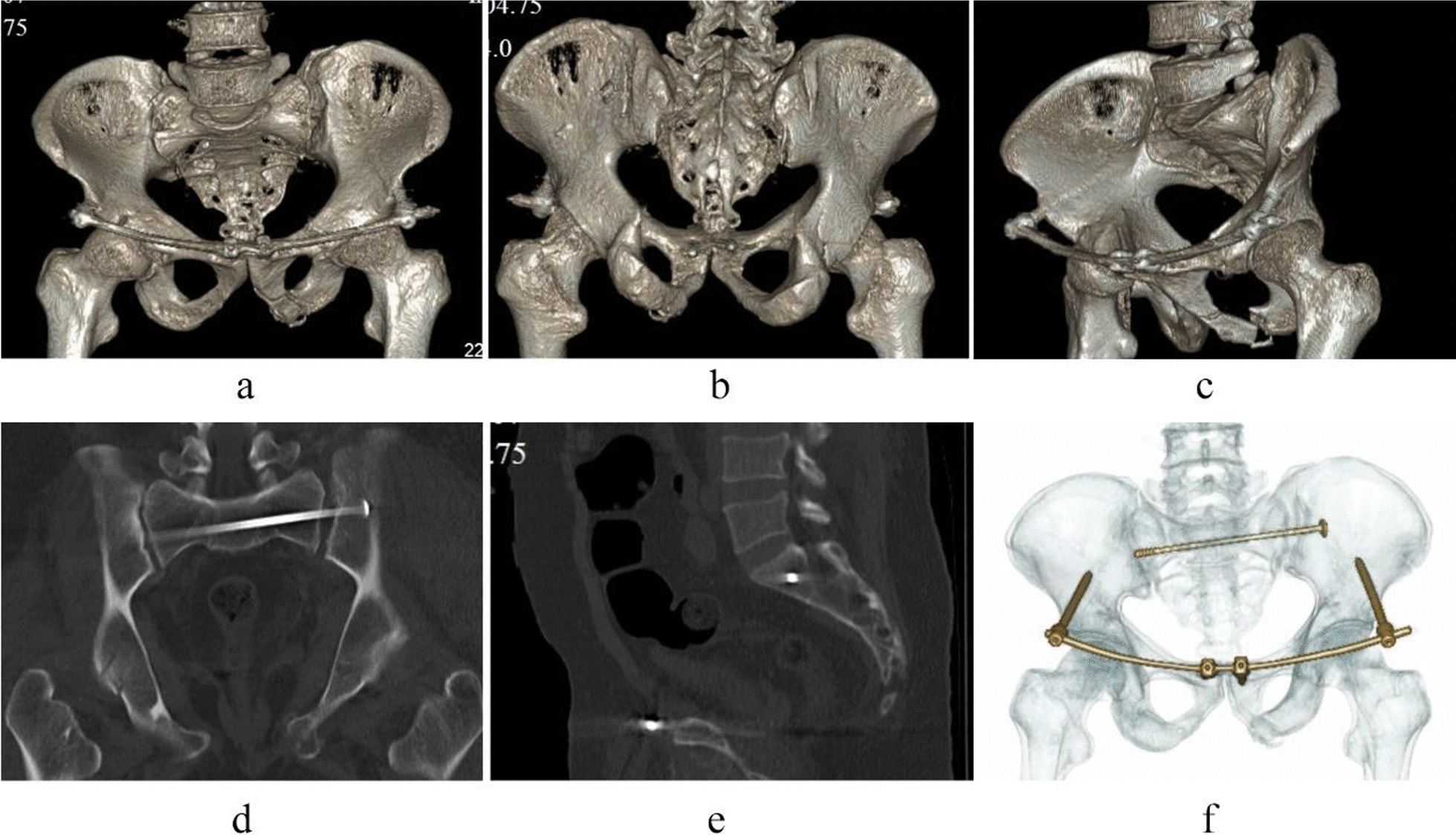
Postoperative CT images of case 3. (**a**–**c**, **f** CT 3D reconstruction image, **d** CT sagittal image, **e** CT coronal slice image.)

Case 3, Liu XX, a 45-year-old male patient, was admitted to hospital because of a car crash injury 24 days earlier. Diagnosis was a pelvic fracture (Tile C2) combined with a left proximal humerus fracture, multiple rib fractures, a left transverse process fracture at the levels of L2–4, a left common peroneal nerve injury, and a left tibiofibular fracture. Preoperative images are presented in Fig. 8. On the second day of admission, the patient underwent closed reduction of the pelvic fracture, including percutaneous cannulated screw internal fixation for the posterior pelvic ring fracture and four-screw-internal fixator system for the anterior pelvic ring fracture. The operation duration was 65 min, with a 30 ml bleeding volume. Postoperative images are shown in Figs. 9 and 10. The patient was discharged after 20 days of hospitalization and followed up for 21 months. The Majeed functional score at the final follow-up was 88 points.

#### Surgical method in group B

The percutaneous sacroiliac screw fixation was performed for posterior pelvic ring injury using the same method as above.

**Fig. 11:**
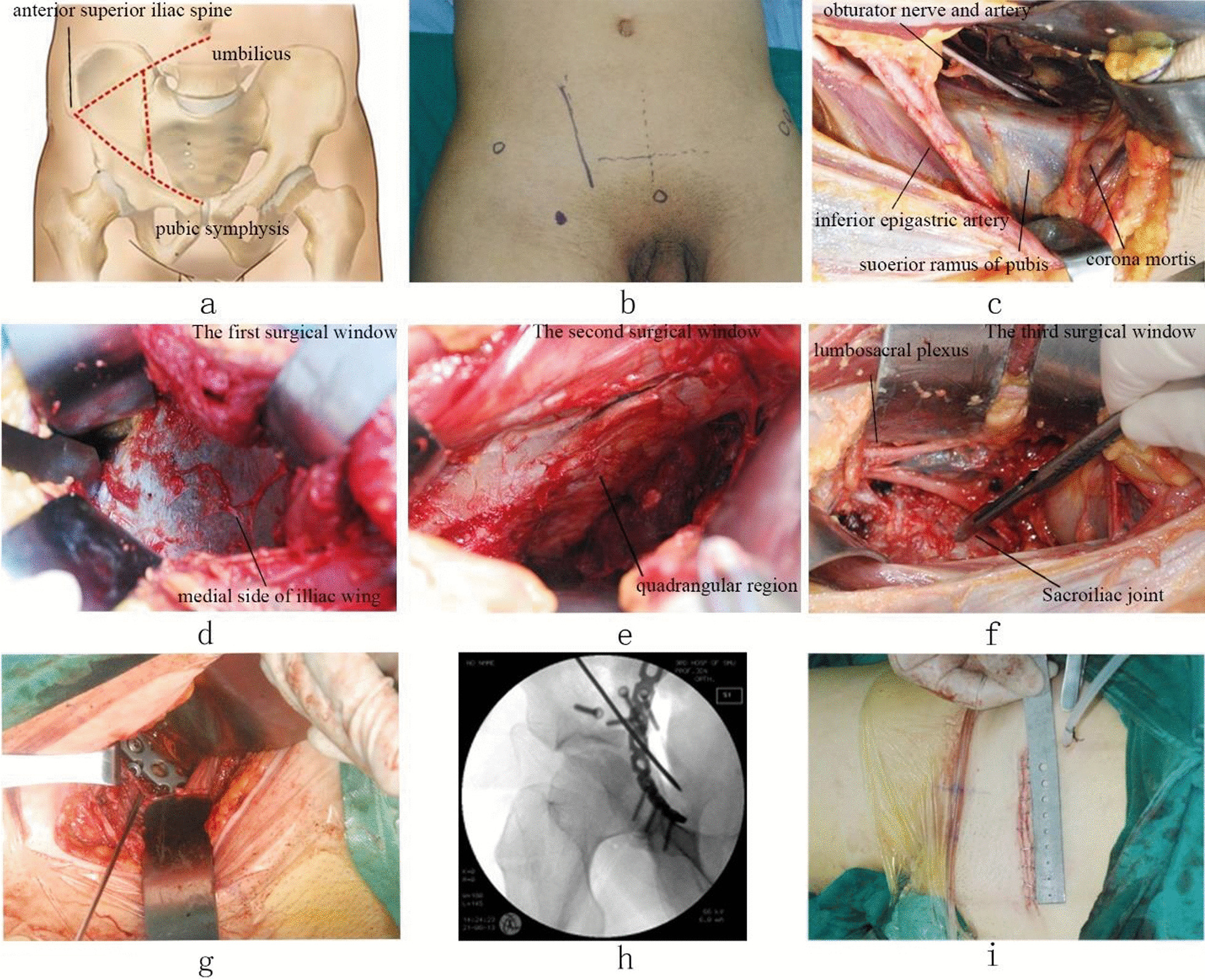
Lateral rectus abdominis approach for plate and screw fixation combined with sacroiliac screw fixation. (**a** Incision design, **b** body surface incision, **c** incision exposure, **d** first operation window, **e** second operation window, **f** third operation window, **g** plate placement, **h** intraoperative fluoroscopic image, **i** postoperative incision.)

The operation was performed via a lateral rectus abdominis approach [6] or modified Stoppa approach [7–9]. The lumbosacral plexus nerve was explored and released first if the patient had a lumbosacral plexus injury. See Fig. 11 for the operation via a lateral rectus abdominis approach.

##### Typical case

**Fig. 12:**
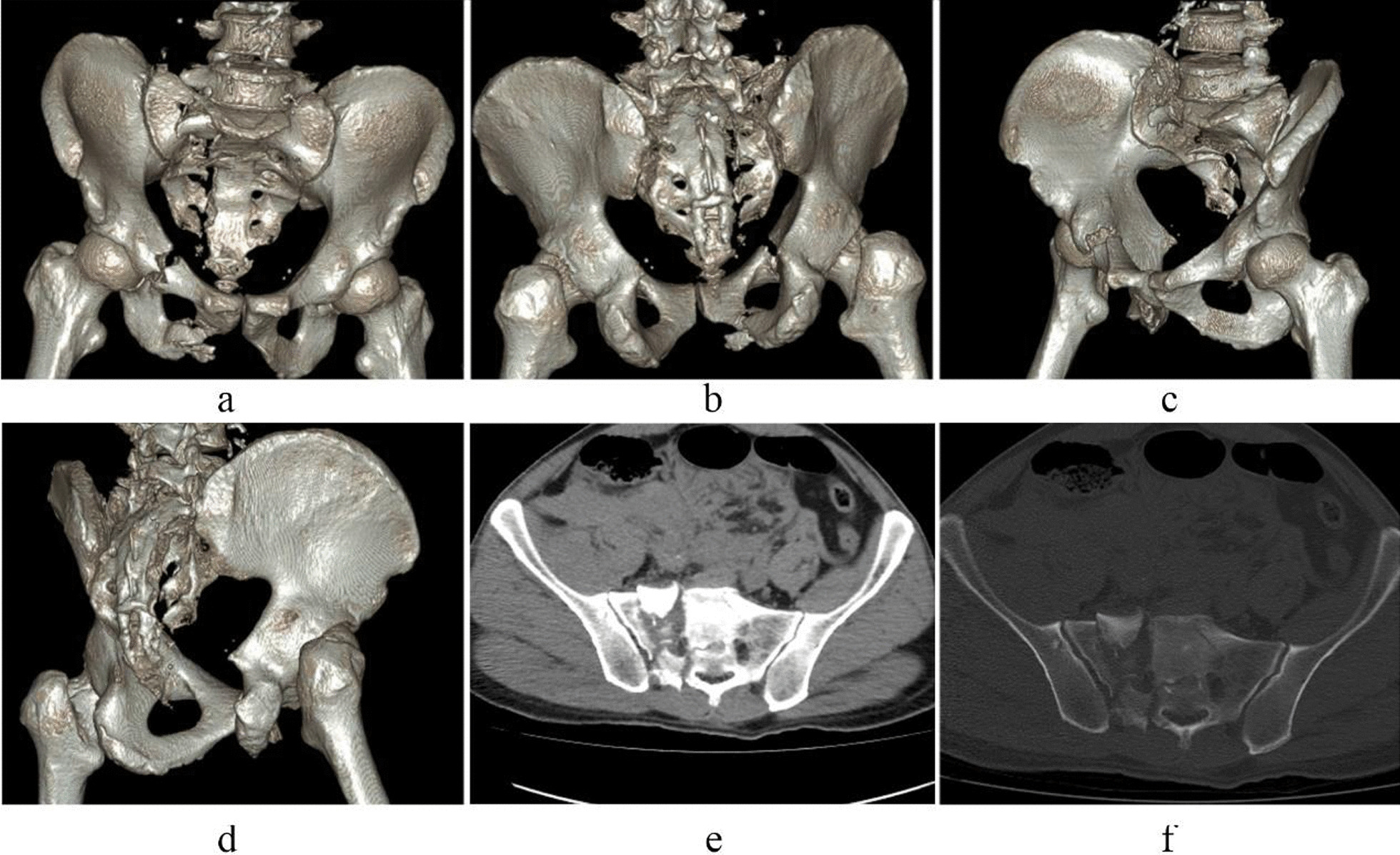
Preoperative CT images of case 4. (**a**–**d** CT 3D reconstruction images, **e**, **f** CT horizontal slice images.)

**Fig. 13:**
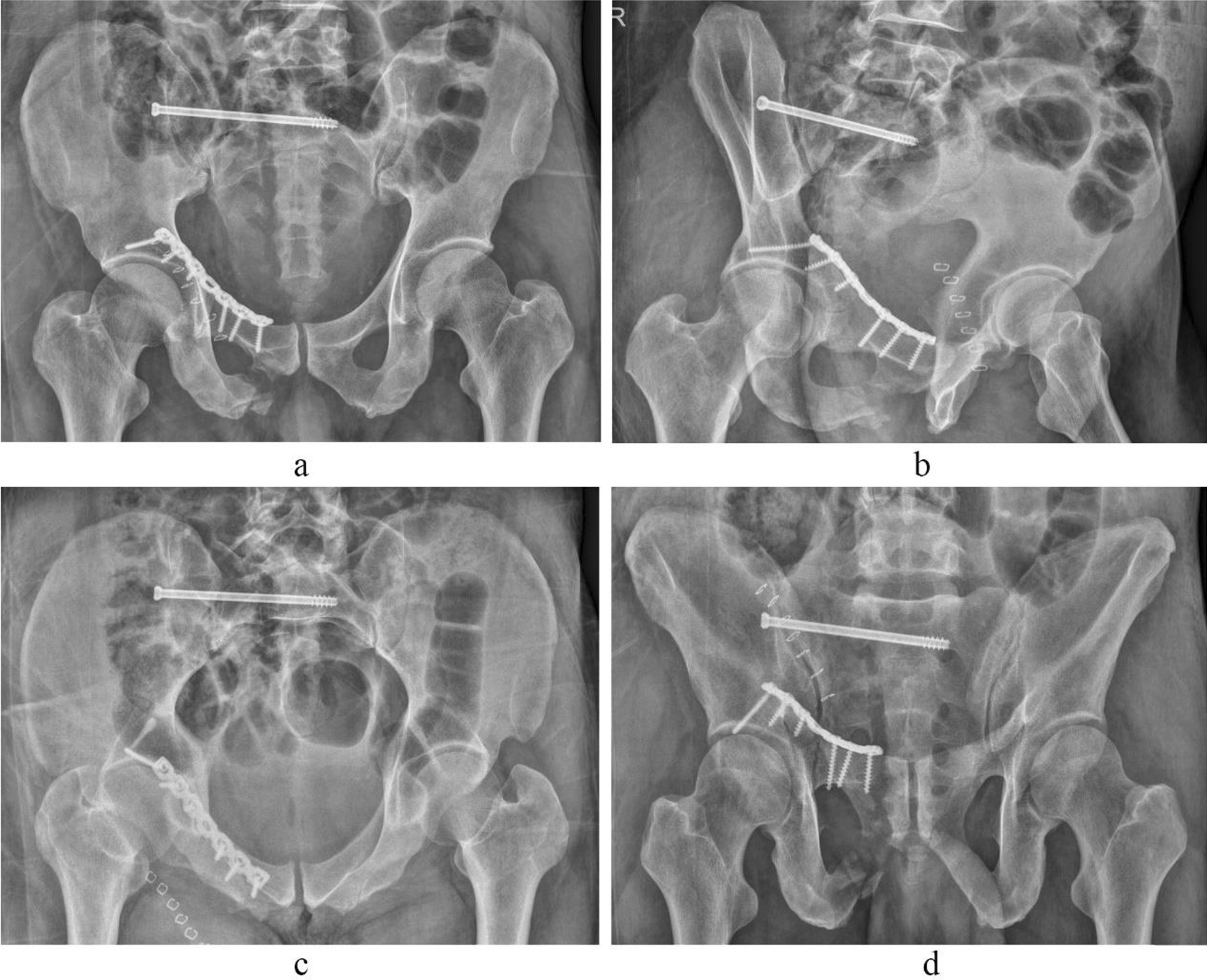
Postoperative X-ray images of case 4. (**a** Pelvic anteroposterior X-ray image, **b** obturator oblique position X-ray image, **c** pelvic inlet X-ray image, **d** pelvic outlet X-ray image.)

Case 4 Zhao XX, a 53-year-old male patient, was admitted to hospital because of a falling injury 6 h earlier. Diagnosis was a pelvic fracture (Tile C1) combined with right transverse process fractures at the levels of L1–5. The preoperative images are presented in Fig. 12. On the sixth day after injury, the patient underwent open reduction of the pelvic fracture via a right lateral rectus abdominis approach, including percutaneous cannulated screw internal fixation for the right sacral fracture and plate and screw internal fixation for the anterior pelvic ring fracture. The operation duration was 105 min, with a 400 ml bleeding volume. The patient was discharged after 17 days of hospitalization. Postoperative images are presented in Fig. 13. The patient was followed up for 24 months, with a Majeed functional score at the final follow-up of 92 points.

### Postoperative treatment and follow-up

At 6 h after either operation, all patients were treated with oral rivaroxaban administration for 2 weeks to prevent deep vein thrombosis of the lower extremities.

During a follow-up period of 1–3 years, the patients were followed up regularly at 1, 2, 3, 6 and 9 months and 1 year after operation, with the follow-up period after one year being every 6 months.

The quality of the reduction of the pelvic fractures was evaluated using the Mata standard [10, 11], while the postoperative functional recovery was evaluated using the Majeed scoring system [12]. Sacral nerve injury recovery was evaluated according to the Gibbons classification [13].

### Statistical methods

All data were analyzed using SPSS22.0 software (IBM Co, USA). The measurement data were comparatively analyzed between groups using independent sample *t*-test. *P* < 0.05 indicated a statistically significant difference.

## Results

None of the patients displayed any complications, such as neurovascular injury, internal fixation loosening or disruption or fracture reduction loss. All fractures healed within 3–6 months. All patients with lumbosacral plexus injury recovered within 6 months after operation. The evaluation indexes are presented in Table 3.

**Table 3:** Case evaluation index

Index	Group A	Group B	*P* value
Time to surgery after injury (d)	6.9 ± 4.0	7.1 ± 4.2	0.809
Operative time (min)	68.2 ± 16.0	109.1 ± 21.8	0.000
Bleeding volume (ml)	37.4 ± 21.4	431.0 ± 254.7	0.000
Transfusion volume (ml)	0	266.7 ± 270.8	0.000
Incision length (cm)	4.5 ± 1.1	9.0 ± 3.8	0.000
Hospital stay (d)	10.2 ± 2.7	20.9 ± 5.7	0.000
Follow-up period (m)	17.9 ± 5.4	20.7 ± 7.2	0.134
Majeed score	89.7 ± 4.6	88.7 ± 4.9	0.527
Matta evaluation rate	100%	100%	0.960
Complication rate	3.7%	23.8%	0.037

Two patients in group A underwent open reduction and lumbosacral plexus surgery through lateral rectus abdominis incision, while the other patients underwent closed reduction and minimally invasive surgery. One patient displayed unilateral lateral femoral cutaneous nerve stimulation symptoms, which resolved 1 month later. The evaluation of the postoperative fracture reduction according to Matta criteria was excellent in 19 cases and good in eight cases, with a combined excellent and good rate of 100%. The Majeed score at the final follow-up was excellent in 22 cases and good in five cases, with a combined excellent and good rate of 100% and a mean score of 89.7 points. The anterior ring internal fixator system was removed in all 27 patients at 3–6 months after the operation.

In group B, 16 patients underwent surgery via a lateral rectus abdominis approach, and five patients underwent surgery via a Stoppa approach. Four patients displayed superficial incision infection, which healed after dressing change for 1 weeks. One patient had femoral nerve injury, which resolved after 3 months. The evaluation of the postoperative fracture reduction according to the Matta criteria was excellent in 20 cases and good in one case, with a combined excellent and good rate of 100%. The Majeed score at the final follow-up was excellent in 16 cases and good in five cases, with a combined excellent and good rate of 100% and a mean score of 88.7 points. The internal fixation plate was removed in 10 patients at 12–18 months after the operation.

There were no significant differences in the time from injury to surgery, postoperative follow-up time, postoperative Matta evaluation or Majeed score between the two groups (all *P* > 0.05) (Table 3). By contrast, there were significant differences in the operative time, incision length, intraoperative bleeding volume, blood transfusion volume, hospital stay and complication rate between the two groups, which were all less in group A (all *P* < 0.05) (Table 3).

## Discussion

### Minimally invasive treatment of pelvic fractures

The anterior and posterior rings in Tile C pelvic fracture are unstable, frequently accompanied by injuries in organs or other parts of the body, and early surgical treatment is very difficult. External fixator fixation has the advantages of creating only a small injury and being a simple operation, but its fixation effect is not reliable, and the incidence of complications, including needle channel infection, loosening and fixation failure, is as high as 12–58% [14], and it is generally used for emergency temporary fixation of pelvic fractures. Traditional open reduction and plate-screw internal fixation is a method with satisfactory reduction and reliable fixation, but it has various disadvantages, including significant surgical trauma, high risk for intraoperative injury, long operative time, large intraoperative bleeding volume and high complication rates [15].

With the development of minimally invasive technologies, minimally invasive closed reduction with percutaneous sacroiliac screw fixation has been commonly used in the treatment of posterior pelvic ring injury. Closed reduction with percutaneous antegrade or retrograde cannulated screw fixation of an anterior ring fracture has been performed in clinical practice, which is suitable for simple pubic fracture or pubic symphysis separation, with a fixation failure of 6–15% in the clinic [16, 17]. However, this method cannot be used to treat comminuted fracture of the anterior pelvic ring. Additionally, the technique requires a long learning curve for operators, which limits its clinical application. In 2009, Kuttner et al. [18] applied an anterior subcutaneous pelvic internal fixator in subcutaneous anterior ring fracture fixation. In 2012, Vaidya et al. [3] called this method INFIX technology; this type of technology being increasingly applied in minimally invasive surgery for anterior pelvic ring fracture. Currently, INFIX is mainly applied in minimally invasive treatment for Tile B pelvic fractures. In this study, we innovatively applied the anterior ring internal fixator system combined sacroiliac screw fixation for Tile C pelvic fractures, the results showed that this method is effective, simple in the minimally invasive treatment for Tile C pelvic fractures.

### Biomechanics and advantages of anterior ring internal fixator system combined with sacroiliac screw fixation in Tile C pelvic fracture treatment

Osterhoff et al. [19, 20] considered that the anterior ring internal fixator has similar translational stability and better rotational stability compared with the external fixator, which is thus a useful method to treat pelvic ring injury. It is believed that the internal fixator system is suitable for the minimally invasive treatment of anterior ring fractures and has achieved good results [21, 22]. Among its advantages are it being a simple operation with minimal trauma and little impact on daily life [23, 24]. Liu et al. [25, 26] performed biomechanical research by using pelvic finite element models and cadaveric specimens to construct two-screw, three-screw and four-screw anterior ring internal fixator systems and a screw-plate system combined with a sacroiliac screw for Tile C3 pelvic fracture fixation. They considered that an anterior ring internal fixator system combined with sacroiliac screw fixation could provide sufficient biomechanical stability for Tile C3 pelvic fracture. The biomechanical stability can be gradually improved with an increasing number of fixation screws, and the biomechanical stability of the four-screw anterior ring internal fixator system combined with sacroiliac screw fixation is similar to that of a plate-screw system combined with sacroiliac screw fixation. In the present study, the internal fixator system combined with sacroiliac screw fixation was innovatively applied in minimally invasive surgery for Tile C pelvic fracture. This has the combined advantages of an anterior ring internal fixator system and percutaneous sacroiliac screw fixation, including minimal invasiveness, being a simple operation and causing less damage, while also providing effective and reliable fracture fixations. Moreover, it also has the advantages of traditional plate-screw fixation, including easy postoperative care and enabled early functional exercise. In the present study, the hospital stay, operative time, intraoperative bleeding volume, blood transfusion volume, incision length and complication rate in group A (anterior ring internal fixator system combined with sacroiliac screw fixation) were significantly better than those in group B (plate-screw system combined with sacroiliac screw fixation), while there were no significant differences in fracture reduction, postoperative function or nerve recovery between the two groups.

### Main complications of pelvic fracture fixation with an anterior ring internal fixator system

The main complications of pelvic fracture fixation with an anterior ring internal fixator system include lateral femoral cutaneous nerve and femoral nerve injuries. Kuttner et al. [18] reported a complication rate of 32%. Vaidya et al. [27] reported that the most common complications are heterotopic ossification (35%), lateral femoral cutaneous nerve stimulation symptoms (30%) and superficial infection (3–4%). Hesse et al. [28] reported six cases of femoral nerve injury. In the present study, no patients displayed complications such as femoral neurovascular injury, heterotopic ossification or local infection in group A, with the complication rate of 3.7% significantly lower than the 23.81% in group B. One patient in group A had unilateral lateral femoral cutaneous nerve stimulation symptoms, which were resolved 1 month later. We believe that most of the complications can be avoided provided that the operation is performed correctly and carefully.

### Sacroiliac screw fixation for posterior pelvic ring injury

Percutaneous sacroiliac screw fixation is a well-established and effective minimally invasive technique for posterior pelvic ring injuries, including sacroiliac joint injury and Dennis I and II sacral fractures, and it is a centric fixation method that passes directly through the fracture ends, thereby providing sufficient stability to effectively resist vertical shear and torsion forces [29, 30]. It has various advantages, including little trauma, less bleeding, shorter operative time and more rapid fracture healing. In the present study, in all patients undergoing sacroiliac screw fixation for posterior pelvic ring fracture, satisfactory reduction and fixation were obtained, achieving good results. The curative effect of sacroiliac screw fixation depends on accurate screw placement [31]. Improper screw placement can readily cause damage to the sacral nerve and presacral vessel injuries, and screw misimplantation can readily lead to nerve root injury, with an incidence of 2–15% [32]. To correctly and safely place the sacroiliac screw requires careful preoperative planning, with considerable requirements placed on the operators’ surgical skills and for auxiliary equipment. In the present study, the surgeons were very skilled with screw placement technology and could safely place a sacroiliac screw within an average of 10 min with the assistance of a C-arm X-ray machine. No complications occurred in any of the patients in the present study. In recent years, with the progress of percutaneous sacroiliac screw placement technology and the application of computer-aided navigation systems, the safety of percutaneous sacroiliac screw internal fixation has been greatly improved, which shortens the operative time and decreases the number of intraoperative X-ray exposures [33, 34].

### Surgical skills

The complication rate after the application of an anterior ring internal fixator system varies greatly in the literature, such that anterior ring internal fixator system safety has been intensively investigated. Validya et al. [3] made clear reported definitive requirements on how to safely place the fixator system, which required that the pedicle screw cap should be 1.5–5.0 cm from the bone surface, the screw should be inserted until the screw cap is just located on the surface of the iliac bone, the connecting rod should be located on the Bikini line after passing through the subcutaneous channel [35], and the excessive length of the connecting rod should be cut off. Merriman et al. [36] proposed that the mean distance between the connecting rod and the vascular bundle should be > 2.2 cm, which can prevent blood vessel and nerve compression.

Apivatthakakul et al. [37] considered that all tissue structures that are at risk of injury are close to the connecting rod, and the risk for the femoral nerve being compressed by the connecting rod is highest. It was suggested that a universal screw should be used for fixation, and a specific space should be left between the screw and the surface of iliac bone, and both ends of the connecting rod should be as short as possible to reduce the risk of lateral femoral cutaneous nerve injury. Osterhoff et al. [38] considered that the safest distance between the connecting rod and the bone surface to be 2 cm, which least interferes with the femoral nerve and blood vessels and lateral femoral cutaneous nerve among others, and also interferes least with the sartorius muscle and the rectus femoris muscle among others in the sitting position.

During the operation, attention should be paid to the difference in pelvic morphology between men and women. The screw insertion position in the iliac region is approximately 5 mm above the anterior inferior iliac spine. The angles between the screw and the coronal, sagittal and horizontal planes are approximately 56°, 26° and 65°, respectively, in men, and 51°, 25° and 59°, respectively, in women. The screw diameter is 7.5 mm, and the screw length is 70–90 mm in men and 60–80 mm in women. The screw insertion position in the pubic region is approximately 10-mm-lateral to the symphysis pubis. Screw insertion is perpendicular into the surface of the pubic bone from top to bottom. The screw diameter is 4.5 mm, and the screw length is 35–45 mm in men and 35 mm in women. The fixation screw can be rapidly and safely inserted into the internal fixator system. Universal screws should be used as fixation screws. The tail part of the screw can be slightly reoriented to facilitate the insertion of the connecting rod and is approximately 10–15 mm away from the bone surface, and the distance between the connecting rod and the bone surface is 20–25 mm. The shape of the connecting rod is pre-bent forward and downward into an arch-shaped configuration, and its length is measured. The connecting rod passes through the deep fascia surface, and the distances between the two ends of the connecting rod and the fixation screws are 5 mm. In the present study, patients displayed no symptom of femoral neurovascular injury, and only one patient had mild anterolateral femoral cutaneous nerve stimulation symptoms. We believe that this method is rapid, safe, reliable and easy to operate based on our experience and careful operation.

With the increase in the number of anterior ring fixation screws, the biomechanical stability of anterior ring internal fixator system combined with sacroiliac screw fixation for Tile C pelvic fracture is gradually improved [25, 26]. In clinical application, the operation with a two-screw internal fixator system combined with sacroiliac screw fixation is simpler and more rapid, because three-screw and four-screw internal fixator systems combined with sacroiliac screw fixation require additional incision and screw placement in the pubic symphysis region, which makes the operation more complex and thus takes longer. For most of the pelvic fractures, a two-screw internal fixator system is sufficient. For unstable anterior ring fractures with pubic symphysis separation and comminuted fracture of the pubic rami, fixation screws can be added into pubic region to assure better biomechanical stability.

### Study limitations

In this study, an anterior ring internal fixator system combined with sacroiliac screw fixation was used to treat Tile C pelvic fracture, which achieved good preliminary results. However, because of the limited number of cases and follow-up period, its clinical adaptability, advantages, disadvantages and long-term effect still need to be followed up and observed for a longer period in more cases.

## Conclusion

An anterior ring internal fixator system combined with sacroiliac screw fixation can effectively treat Tile C pelvic fracture, which has various advantages, including minimal invasiveness, simple operation, short operative time, safe and reliable features, less complications, short hospital stay and a good curative effect.

## Data Availability

The datasets used and analyzed in the study are available on request to the corresponding author.

## Abbreviations

CT: Computed tomography
INFIX: Internal fixator
